# What Makes for the Most Intense Regrets? Comparing the Effects of Several Theoretical Predictors of Regret Intensity

**DOI:** 10.3389/fpsyg.2016.01941

**Published:** 2016-12-15

**Authors:** Andy Towers, Matt N. Williams, Stephen R. Hill, Michael C. Philipp, Ross Flett

**Affiliations:** ^1^School of Public Health, Massey UniversityPalmerston North, New Zealand; ^2^School of Psychology, Massey UniversityAuckland, New Zealand; ^3^School of Psychology, Massey UniversityPalmerston North, New Zealand

**Keywords:** regret, justification, inaction, action, Bayesian

## Abstract

Several theories have been proposed to account for variation in the intensity of life regrets. Variables hypothesized to affect the intensity of regret include: whether the regretted decision was an action or an inaction, the degree to which the decision was justified, and the life domain of the regret. No previous study has compared the effects of these key predictors in a single model in order to identify which are most strongly associated with the intensity of life regret. In this study, respondents (*N* = 500) to a postal survey answered questions concerning the nature of their greatest life regret. A Bayesian regression analysis suggested that regret intensity was greater for—in order of importance—decisions that breached participants’ personal life rules, decisions in social life domains than non-social domains, and decisions that lacked an explicit justification. Although regrets of inaction were more frequent than regrets of action, regrets relating to actions were slightly more intense.

## Introduction

Our ability to make decisions that in retrospect appear breathtakingly misguided is a trait spanning social and cultural divides, and crossing gender and age barriers. For example, despite protests from wise counsel, Priam chose to open the gates of Troy to the gift of a large wooden horse. The president of the Western Union Telegraph Company decided to forgo Alexander Graham Bell’s offer of the patent rights on the “telephone” which he considered an expensive “electrical toy.” After sitting through a live hour-long audition, Decca Record executives decided not to offer a contract to a young quartet called “The Beatles.” Each of these decisions no doubt resulted in an important emotional response: *regret*. Regret is very common (Shimanoff, 1984; Landman, 1987), and is felt when the outcome of a decision is worse than the outcome of an option foregone (Zeelenberg, 1999). Research over the last several decades has produced several competing theories seeking to predict what makes for the most intense regrets; each theory suggests unique explanations for the intensity of regret. Despite rising interest in regret research, no study has to date comprehensively compared the key predictors of regret from competing theories in a single statistical model in order to ascertain their relative importance in affecting the intensity of our life regrets. This study aims is to fill this gap by focusing on three distinct theories that have identified competing predictors of regret.

### Three Theories of Regret

The temporal theory of regret (Gilovich and Medvec, 1994, 1995) is one of the most well-known regret theories and suggests that lifespan changes in regret intensity are driven by the *nature* of the regrettable decision itself (i.e., whether the regret relates to an action or an inaction). Specifically, the temporal theory of regret suggests that “[a]ctions produce greater regret in the short-term, whereas inactions generate more regret in the long run” (Gilovich and Medvec, 1994, p. 361). Gilovich and Medvec (1995) propose a variety of mechanisms that might explain why regret for inactions might increase over time compared to regret for actions, including the idea that outcomes resulting from mistaken actions can be ameliorated by opportunities afforded by the passage of time, whereas there may not be similar opportunities to correct the outcomes of regretted inactions.

Another key contemporary theory of regret is decision-justification theory (Connolly and Zeelenberg, 2002), which identifies the underlying decision *rationale* as key for regret generation. Similarly to other theories of regret, decision-justification theory proposes that one key influence on regret is the outcome of the decision: We feel more regret over decisions whose outcomes compare poorly to those of options foregone. However, decision-justification theory suggests furthermore that the intensity of regret felt also depends on the degree to which the individual identifies the decision as *justifiable:* A decision that results in a poor outcome will cause less regret if the decision was, in retrospect, still justified.

In contrast to both the temporal theory of regret and decision-justification theory, a third “belonging” theory of regret (Morrison et al., 2012) identifies the *context* of the regrettable decision as foundational in driving regret intensity. Specifically, Morrison et al. (2012) found that regrets in domains particularly relevant to our senses of social belonging (e.g., romantic or family domains) are more intensely felt than those in less social domains (e.g., work, education), suggesting that an individual experiences more regret when a poor decision is perceived to represent a threat to their sense of social belonging.

In sum, each of these three theories incorporates unique hypotheses concerning the key predictors of regret intensity; our study thus seeks to test these hypotheses. In addition, there is little evidence in the extant literature that the various predictors of regret specified in these three theories have been compared with each other in a single statistical model in order to assess their relative importance in generating regret intensity. Our study thus seeks to simultaneously assess the effects of these various predictors of regret intensity (whether a regret relates to an action or inaction, how justified the original decision was, and whether a regretted decision was in a social domain), and thereby determine which are the strongest drivers of regret intensity.

### Testing an Extension to Decision-Justification Theory: Implicit Justifications

As noted above, one theory tested in this study is Connolly and Zeelenberg’s (2002) decision-justification theory. Connolly and Zeelenberg note, however, that little is known regarding the nature of justification generation. They suggest that one particularly effective form of justification is the presence of “a careful, competent decision” (Connolly and Zeelenberg, 2002, p. 215)”. However, justifications need not always be rational or explicit: Research also shows that justifications based on our implicit personal values or life-rules affect our experience of regret (Seta et al., 2001; Lönnqvist et al., 2006). This finding reflects core notions in theories of decision-making—like the theory of Regulatory Fit (Higgins, 2000; Camacho et al., 2003)—that maintain that consistency with our *implicit* values is an important factor in decision making. It is therefore feasible that decision justifications present themselves in two forms: *explicit* and *implicit.* To this end it seems likely that explicit axiom-based justifications, as described by Connolly and Zeelenberg (2002) shield one from regret, but that decisions lacking an explicit justification might still incur little regret if they were deemed consistent with an individual’s values or rules (i.e., a justification that is *implicit* in the sense that it stems from the consistency of an action or inaction with one’s individual life rules). We therefore hypothesized that regretted decisions that were consistent with participant’s personal life rules would be less intensely regretted.

### A Methodological Issue: Regret Intensity

An important issue with respect to the theories mentioned above is that while the theories discussed focus on explaining variation in the *intensity* of regret, much of the existing empirical research concerning these theories has actually assessed the *frequency* with which reported regrets fall into particular categories—rather than explicitly measuring regret intensity. For example, Gilovich and Medvec’s (1994) studies analyzed the frequency with which participants’ most greatly regretted decisions fell into particular categories (e.g., action/inaction), rather than directly measuring or analyzing regret intensity. More recent studies similarly have often focused on the frequency with which participants’ regrets fall into particular categories or life domains (e.g., Roese et al., 2009; Morrison and Roese, 2011; but c.f. Wilkinson et al., 2015). Emotional frequency and intensity are distinct processes underlying the experience of affect (Diener et al., 1985; Schimmack and Diener, 1997) and they are not necessarily highly correlated: Frequent emotions (e.g., happiness) may not necessarily be intense, while infrequent emotions (e.g., rage) may be very intense. Studies assessing regret frequency as a proxy for regret intensity are therefore potentially misleading, and this may explain why the few studies directly distinguishing regret frequency and intensity found inaction regrets to be more frequently reported but no more *intense* than action regrets (Feldman et al., 1999; Bonnefon and Zhang, 2008). This study will therefore focus on reported regret intensity (rather than frequency) in order to provide conclusions as to the key drivers of regret intensity across the lifespan.

### Aims and Predictions

In this study, we seek to test the temporal, decision-justification, and belonging theories of regret so as to determine which characteristics associated with a regretted decision are most strongly associated with the intensity of regret. Predictions implied by these theories have been tested in the past, but each theory has generally been tested in isolation. We also deliberately focus on studying the *intensity* of regretted decisions as opposed to simply the *frequency* with which regretted decisions of particular types are reported. Finally, we explore the effects on regret intensity of different forms of justification; namely, both explicit justification and implicit justification (i.e., whether the regrettable decision was consistent with the individual’s implicit life rules).

The following predictions were tested in relation to participants’ self-reported greatest lifetime regrets:

(1) According to the temporal theory of regrets, participants whose greatest lifetime regrets are inactions should report greater regret intensity.
(2) Also according to the temporal theory of regret, the action/inaction variable and the length of time since the regretted decision should have an interactive effect on regret intensity. Specifically, the longer the time since the decision, the greater the difference between action and inaction regrets should be (with inactions resulting in higher regret intensity).
(3) As per decision-justification theory, regretted decisions for which the participants reported greater explicit justification should be associated with *lower* regret intensity.
(4) As an extension of decision-justification theory, regretted decisions that were consistent with participants’ personal life rules (i.e., that had an implicit justification) should be associated with *lower* regret intensity. Conversely, regrets that contradicted participants’ life rules should be associated with higher regret intensity.
(5) As per the belonging theory of regret, regretted decisions in social domains should be associated with *greater* regret intensity than regrets in non-social domains.

## Materials and Methods

### Participants

Three thousand individuals were randomly selected from the New Zealand electoral roll and invited to take part in a postal survey. The initial participant pool size of 3,000 was chosen on the basis that this number was considered sufficient to ensure the return of at least several 100 completed surveys for analysis.

A total of 677 surveys were returned. Of these, 177 did not contain responses to any of the items analyzed in this study, and were excluded from all analyses reported here. A large part of this apparent missing data was due to a screening item early in the questionnaire that asked: “Looking back on your life what level of regret would you say you have?,” with responses on a 5-item rating scale with endpoints of “No regrets” and “Many regrets.” The 126 participants who responded “No regrets” to this item were directed to skip through to a later portion of the questionnaire, bypassing the items examined here. These 126 surveys comprised the majority of the 177 without responses to any of the items analyzed in this study.

Within the resulting final sample of *N* = 500, participants ranged in age from 18 to 87 years, *M* = 47.3, *SD* = 15.2. Sixty-eight percent were female. In terms of ethnicity, the largest groups were Europeans/New Zealand Europeans (82%) and M
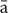
ori (7%). There was great diversity in education level: 48% of the sample held only a high school qualification or below, with 24% having a trade certificate or diploma, and 28% a university degree or diploma.

### Procedure

Once the 3,000 participants had been randomly selected from the electoral roll, a three-stage postal survey process was used in order to increase response rates. Based on Dillman’s (2000) multi-stage tailored design method, the three stages in the current study consisted of (1) an initial letter of invitation to participate in a study described as looking “at the development of life regrets,” (2) a second introduction letter, the postal questionnaire and free-post return envelope, and (3) a reminder postcard.

This study was carried out in accordance with the recommendations of the Massey University code of ethical conduct for research, teaching and evaluations involving human participants with written informed consent from all subjects. All subjects gave written informed consent in accordance with the Declaration of Helsinki. The protocol was approved by the Massey University Human Ethics Committee.

### Measures

Participants were asked to describe (in writing) their greatest life-time regret using the following specific prompt “*Please describe in the space below the specific event or decision that is the single greatest regret from your entire lifetime.”* They were provided with seven lines on an A4 page to write their description. Once they had completed this item, all subsequent questions were asked in relation to this specified regret. The provided written descriptions were generally fairly brief, *M* = 27 words, *SD* = 26 (excluding one outlier comprising a multi-page description).

#### Regret Intensity

In relation to their greatest life-time regret, participants were asked “*How intense are your feelings of regret?*” and were required to circle one number on a 9-point rating scale with five graduated anchor points (1 “I don’t regret it much,” 3 “I regret it somewhat,” 5 “I regret it quite a bit,” 7 “I regret it a lot,” and 9 “I regret it immensely”).

#### Coding Regrets as Actions or Inactions

Participants’ open-ended responses describing their greatest regrets underwent third-party coding to ascertain whether the regret involved action or inaction. Two independent coders, blind to the nature of the research, were asked to code each regret as reflecting either an action or an inaction. Where the coders were unable to identify the regret as stemming from an action or an inaction, they coded the response as indeterminate. These indeterminate responses were subsequently treated as missing data in substantive analyses. Interrater reliability was high, with 90% agreement and Cohen’s κ = 0.86, 95% CI [0.82, 0.91], suggesting excellent agreement according to the guidelines of Fleiss et al. (2013).

#### Coding of Regrets as Social or Non-social

Two further independent coders, also blind to the nature of the research, separately coded each regret as reflecting 1 of 13 life domains similar to those used by Roese and Summerville (2005). Regret domains were subsequently collapsed into two main categories: *Social* regrets (regrets concerning intimate relationships, family, parenting, and friendships), and *non-social* regrets (regrets concerning education, occupation, finance, health and self-care, leisure, travel/moves, and the self). Regrets spanning multiple domains were treated as missing data in the sense that it was unclear if they were primarily social or non-social in nature, as were other regrets that could not be classified within the 13 domains mentioned. Any inter-rater disagreement during coding was settled by the judgment of the first author. Interrater reliability was again high, with 83% agreement on the overarching social/non-social distinction, and Cohen’s κ = 0.91, 95% CI [0.87, 0.95].

#### Time Since Regret Occurred

Participants were asked to “*Please indicate in the space below how long ago (approximately) this decision or event took place*,” with spaces given to write the number of months and years.

#### Level of Explicit Justification

To assess whether participants had identified any explicit justification for their regretted decision they were asked; “*At the time it happened, how justified was the event or decision?*” Response was on a 5-point rating scale ranging from 1 (“Not justified”) to 5 (“Completely justified”).

#### Contradiction of Life Rules (Implicit Justification)

Participants were asked to read the following preface in order to facilitate consideration of a more implicit (i.e., value-based) justification for their decision: “*Some people have personal rules, or life philosophies, that often help guide what they do. For example, some people believe that you should always think of family needs before your own, or some think that the most important thing in life is to be nice to people. Take a minute to think about what some of your most important personal rules are. What rules help guide your decision-making? Try to choose what you believe are some of your most important personal rules. Now that you have them in mind, compare them to the decision or event that you regret. Do you feel that the decision you made or event you experienced contradicts any of your personal rules?*” In response to this question, participants could either tick “No it does not” or “Yes it does.”

#### Additional Measures

The study reported here formed part of a larger project exploring the multiple determinants of regret across the lifespan by authors 1, 3, and 5. Additional data collected but not relevant to the focus of this manuscript included information about participants’ greatest short-term (as opposed to long-term) regrets; the impact of participants’ regretted decisions; the frequency with which they thought about their regrets; the extent to which their regrets prompted each of 15 other emotions; the degree of responsibility they felt in relation to their regrets; the extent to which they felt their decisions were justified (in retrospect, as opposed to at the time); and whether their justifications were based on personal beliefs or situational factors. Participants also completed the brief preference for consistency scale (Cialdini et al., 1995).

### Data Analysis: Bayesian Approach

Null hypothesis significance testing—the dominant data analysis framework in psychology—has many well-established problems (for reviews see Cohen, 1994; Falk and Greenbaum, 1995; Gigerenzer et al., 2004; Wagenmakers, 2007). In this study we use Bayesian estimation (see Kruschke, 2010 for an introduction). Bayesian estimation has three main advantages relevant to the current study: Firstly, unlike null hypothesis significance testing—or any frequentist method—it allows us to directly calculate and report the probability that a particular hypothesis is true (e.g., the probability that a true parameter is positive, given the data observed, *P*(β > 0 | Data)). Secondly, it also allows us to directly report the probability that a particular parameter falls in a specific region (traditional frequentist confidence intervals cannot do this; see Morey et al., 2015). In this study we report the 95% highest probability density (HPD) interval for each parameter. Finally, Bayesian estimation allows us to take into account *prior* information: For example, we know that in psychology, most effects are relatively small in size: Taking this pre-existing information into account helps us estimate parameters more accurately.

In this study, we used a similar prior for all estimated effects (e.g., regression coefficients, mean differences, correlations). This was a normal distribution with mean zero and standard deviation of 0.31, but scaled to apply to the *standardized* effect size for each parameter (i.e., the correlation for a bivariate analysis or partial correlation for a multiple regression analysis). This prior is based on the finding of a meta-meta-analysis of over 25,000 studies in social psychology (Richard et al., 2003), which reported a mean absolute effect size of *r* = 0.21. The prior of *N*(0, 0.31^2^) effectively states that there is 50% prior probability that the standardized effect size will be of absolute magnitude of 0.21 or greater, corresponding with Richard et al.’s (2003) finding. This is an *informative* prior: All else being equal, it will tend to make our findings more conservative (shrinking our point and interval estimates slightly toward zero). The basic framework for using this approach to prior setting is described in Williams (2016). Non-informative priors were set on nuisance parameters (error variances and intercepts).

In terms of computation, data analysis was completed in R version 3.1.1 (R Core Team, 2014) using the MCMCpack package (Martin et al., 2011). With regard to item missingness, in addition to the 177 excluded surveys with no responses on the study variables, there were another 71 surveys with a response to at least one of the study variables, but with missing data (or an unclassifiable response) to one or more items. Multiple imputation (across five datasets) was used to impute the resulting missing data points in the mice package (Buuren and Groothuis-Oudshoorn, 2011). Each Bayesian analysis was conducted on each of the five imputed datasets, with inferential statistics (e.g., posterior means, highest posterior density intervals, etc.) calculated based on the pooled posterior distributions across all the imputed datasets.

### Open Data and Analysis Scripts

Open data and analysis scripts for this study are available at https://github.com/mattnw/Towers-et-al-Regret.

## Results

### Descriptive Statistics

Despite participants being specifically asked about their greatest lifetime regrets, there was considerable variation in regret intensity. The regret intensity ratings traversed the entirety of the possible scale range (1–9), with a mean of 5.97 (*SD* = 2.24). The time elapsed since participants’ greatest regrets also varied widely, with a mean of 21.77 years (*SD* = 15.09). The mean of 2.80 for the explicit justification variable (*SD* = 1.31) was near the midpoint of 3 (a regretted decision that was “somewhat” justified). In terms of the dichotomous explanatory variables used in this study, participants’ regrets were primarily in non-social (62%) rather than social domains, and the act or decision they regretted typically contradicted their personal life rules (57%). Slightly more of the regrets were for inactions (54%) as opposed to actions.

### Bivariate Analyses

The following analyses describe the relationship between each of the individual explanatory variables in this study and the response variable (regret intensity). Firstly, the relationships between regret intensity and the three dichotomous explanatory variables were assessed. These dichotomous variables were whether the regret was for an action or for inaction; whether the life domain of the regret was social or non-social; and whether the regret contradicted the participant’s personal life rules. The results are displayed in **Table 1**. Interestingly, despite participants being more likely to report inaction regrets as their greatest lifetime regrets, the intensity of action regrets was higher. Participants also reported greater regret intensity when the regret was in a social life domain and when the regret contradicted their life rules.

**Table 1:** Relationships between dichotomous variables and regret intensity.

	Yes			No			Mean difference (sample)	95% highest probability density (HPD)	Cohen’s *d*	P(δ > 0 | Data)
	*M*	*SD*	*n*	*M*	*SD*	*n*				
Action regret	6.27	2.21	228	5.71	2.23	272	0.57	0.16, 0.95	0.25	0.996
Social regret	6.47	2.12	192	5.65	2.26	308	0.81	0.37, 1.23	0.37	>0.999
Regret contradicted life rules	6.41	2.07	285	5.38	2.33	215	1.04	0.63, 1.41	0.47	>0.999

*Possible scale range for regret intensity: 1–9. All values (including subsample *n* sizes) are averaged across five multiply imputed datasets. Means, standard deviations, mean differences and Cohen’s *d* statistics reported here are sample statistics; HPD and *P*(δ > 0 | Data) are based on Bayesian estimation with a prior of *N*(0, 0.31^2^) scaled in terms of the standardized *r* effect size.*

Participants who reported higher levels of explicit justification reported lower regret intensity, *r* = -0.13, with strong evidence that the true correlation in the population was negative, 95% HPD [-0.22, -0.05], *P*(ρ < 0 | Data) = 0.999. On the other hand, time since the regret had virtually no relationship with intensity in the sample, *r* = 0.01, 95% HPD [-0.08, 0.09], with the direction of the true relationship remaining unclear, *P*(ρ > 0 | Data) = 0.564.

### Multiple Regression Analysis

The analysis that follows assesses how the relationships between the explanatory variables and regret intensity change when their effects are assessed simultaneously. All explanatory variables were entered simultaneously in this model, the coefficients of which are shown in **Table 2**. It is immediately apparent that the effects of action/inaction and level of explicit justification are attenuated when the other explanatory variables are controlled for. The model suggests that when the other predictors are held at zero, the mean difference in regret intensity between an action and an inaction is just 0.08 units on the regret intensity scale (this scale ranging from 1 to 9). As in the bivariate analysis, it was actions that were associated with higher regret, although there was a great deal of uncertainty about this conclusion, *P*(β > 0 | Data) = 0.604.

**Table 2:** Multiple regression model with regret intensity as response variable.

Coefficient	Un-standardized	95% HPD lower	95% HPD upper	Standardized	*P* (β > 0 | Data)
Intercept	5.51	4.78	6.21		>0.999
Regret of action (inaction = 0, action = 1)	0.08	-0.55	0.73	0.02	0.604
Time since regret (years)	0.00	-0.02	0.02	0.01	0.540
Action/inaction^∗^Time	0.02	-0.01	0.04	0.10	0.901
Level of explicit justification for regret	-0.16	-0.31	-0.01	-0.09	0.016
Contradiction of implicit life rules (no = 0, yes = 1)	0.82	0.44	1.22	0.18	>0.999
Social regret (no = 0, yes = 1)	0.62	0.19	1.04	0.13	0.998

*Possible scale range for regret intensity: 1–9. All values are averaged across five multiply imputed datasets. All statistics reported here based on Bayesian estimation with a prior of *N*(0, 0.31^2^) scaled in terms of the standardized *r* effect size.*

The two explanatory variables with the largest effects were whether or not the regret was in a social domain, and whether it contradicted the participant’s life rules. On the other hand, explicit justification level had a small effect: An increase of one unit on the 1–5 explicit justification scale was associated with a reduction of just 0.16 units on the intensity scale, although there was sufficient evidence that the true parameter was negative, *P*(β < 0 | Data) = 0.984.

Importantly, the direction of the estimated coefficient for the interaction between action/inaction and time was not in accordance with the predictions of the temporal theory of regret. The point estimate of the coefficient was positive, suggesting that the “effect” of a regret being one of action was *larger* (and more positive) when more time had elapsed since the regretted decision. This said, the *P*(β > 0 | Data) value of 0.901 implies that while there is relatively high probability that the true direction of this effect is positive, there does remain some non-trivial uncertainty.

## Discussion

This study was the first to concurrently assess the influence of multiple regret enhancing mechanisms from the temporal, decision-justification and belonging theories on the intensity of experienced life-regret in a sample of adults. In accordance with the temporal theory of regret (Gilovich and Medvec, 1994, 1995), participants reported greater frequency of lifetime regrets of inaction rather than action, but our results concerning the actual intensity of these regrets do not support the predictions outlined by the temporal theory. Firstly, we found no evidence to support the assertion of Gilovich and Medvec (1995, p. 381) that “when people look back on their lives, it seems to be their regrettable failures to act [inactions] that stand out and cause greater grief.” Rather, the effect of action/inaction was small, and it was *actions* that were associated with greater regret intensity. Furthermore, in line with previous studies (Feldman et al., 1999; Bonnefon and Zhang, 2008) we found no evidence to support the hypothesis that the passing of time leads to an increase in regret intensity for inactions and a converse reduction in intensity for actions.

The second theory tested in our study was the decision-justification theory of regret (Connolly and Zeelenberg, 2002). In accordance with this theory, explicit justification level was negatively related to regret intensity: Participants who felt that they were more justified in their regretted decisions tended to report less intense regret. This finding was in accordance with previous studies showing a negative relationship between level of justification and regret intensity (Inman and Zeelenberg, 2002; Reb and Connolly, 2010). This said, the relationship between explicit justification and regret intensity in our study was small.

On the other hand, participants who reported that their regretted decision was consistent with their personal life rules (our operationalization of an *implicit* justification) reported less intense regret, with a standardized coefficient about twice the size of that for explicit justification level (although this effect was still not very large in size). This finding suggests a potential modification of decision-justification theory such that both explicit and implicit justifications might be theorized to ameliorate regret intensity. Of course, there could be other ways to operationalize the concept of an implicit justification in future research. For example, behavioral measures of implicit attitudes (e.g., Fazio and Olson, 2003) toward particular types of regretted behaviors could be used to examine how strongly individuals associate their regretted behaviors with the concept of “justifiable.” It would then be possible to test whether participants who implicitly regard their behavior as justifiable experience less intense regret.

Finally, the belonging theory of regret (Morrison et al., 2012) received some support in our study. Congruent with the predictions by Morrison et al. (2012) regrets experienced in social domains were more intense than those experienced in non-social domains (although the effect size was not very large). This finding supports Morrison et al.’s (2012) theory that the extent to which a regretted decision constitutes a threat to an individual’s sense of belonging (e.g., because it might compromise his or her interpersonal relationships) has an important influence on the intensity of regret felt. Morrison’s theory may be valuable in that—to a greater extent than the temporal and decision-justification theories—it takes into account how a regretted decision impacts and relates to an individual’s broader situational and social context.

This study did have some limitations of scope, most notably in that we were obviously to unable to test all contemporary theories of regret. One example of such a theory is the “opportunity principle” (Roese and Summerville, 2005), which suggests that people feel the most intense regret in relation to regretted actions or inactions that they have an ongoing (but untaken) opportunity to correct. On the other hand, the “lost opportunity principle” (Beike et al., 2009) suggests that regret is greatest for *lost* opportunities: undesirable outcomes that could have been prevented in the past, but that now can no longer be remedied. We did not ask our participants about the capacity that they had to correct their regrets in the past or at the time of data collection, and could not test these theories. Furthermore, we did not present analyses of all of the variables contained within the study questionnaire: This study reports part of a larger project, with future reports planned on topics such as the specific type of emotion (e.g., hot, wistful) felt in relation to regretted decisions. Finally, we did not examine how individual difference variables might moderate the effect of the theoretical predictors of regret that we studied. For example, Feeney et al. (2005) reported that participants high in self-esteem were more likely to identify a regret of inaction as their greatest lifetime regret than were participants low in self-esteem. Feeney et al. (2005) suggested that this might indicate that experiencing regret for inactions is self-enhancing.

The study also had some limitations of design. Although the sample initially invited to take part was selected randomly from the population of adult New Zealanders, the modest response rate means that the sample was not truly random. Furthermore, the theoretical propositions considered in this paper relate to causal influences on regret, but the study’s cross-sectional and correlational design makes it difficult to draw firm causal conclusions. Finally, the study used only self-report data, in effect assuming that participants were both willing and able to provide accurate information about their emotions and behavior. Of course, all three of these limitations are common to much research in this area and cannot be easily circumvented.

## Conclusion

The current study identified the context of regret (social vs. not) and the presence of an implicit decision justification as vital to understanding the intensity of our life regrets. These findings provides support for the belonging theory of regret (Morrison et al., 2012), and for a modified version of the decision-justification theory of regret (Connolly and Zeelenberg, 2002). However, the results of this study offer no support to a third theory (the temporal theory of regret) and strongly suggest that the commonly held notion that inaction regrets increase and action regrets decrease in intensity across the lifespan is in fact an artifact of empirical studies analyzing regret *frequency* rather than regret *intensity*. Future research concerning the nature of regret intensity requires the development of new theories that consider the context of regret and the nature of the justification (or lack thereof) underpinning our regrettable decisions.

## Author Contributions

AT, SH, and RF designed the study. AT collected data. MW conducted data analyses, with assistance from MP. AT and MW drafted the manuscript. All authors contributed to revising the manuscript for important intellectual content, give final approval for this version to be published, and agree to be accountable for all aspects of the work.

## Conflict of Interest Statement

The authors declare that the research was conducted in the absence of any commercial or financial relationships that could be construed as a potential conflict of interest.
